# Hospital Almoners' Council

**Published:** 1913-12-20

**Authors:** 


### Hospital Almoners' Council.

Sir Malcolm Morris, Vice-President, took the chair at the annual meeting of the Hospital Almoners' Council last week. The Secretary, Mr. E. S. Kemp, stated that fifteen candidates for almoners' posts were in course of training. He referred to the appointment of an in-patient almoner at Great Ormond Street Hospital for Sick Children, and remarked on the numerous appointments of assistant almoners, as evidence of appreciation of the system on the part of those hospitals which had adopted it. In moving the adoption of the annual report, Sir Malcolm Morris said that, after forty-eight years' experience of hospitals, he was convinced that there was no more important work being done in London than that of the almoners in supplementing the medical treatment provided by the hospitals. He pointed out that unless the work of hospitals is linked up with that of charities outside their walls patients cannot be enabled to benefit fully by the treatment they receive. He referred to the necessity of careful training for the work as provided by the Council. He also said that the public knew far too little of the movement, and urged that it should be brought into much greater prominence, towards which he himself would be glad to assist in any way.

